# An explorative study on respiratory health among operators working in polymer additive manufacturing

**DOI:** 10.3389/fpubh.2023.1148974

**Published:** 2023-04-17

**Authors:** Ann-Charlotte Almstrand, Anna Bredberg, Gunilla Runström Eden, Helen Karlsson, Maria Assenhöj, Hatice Koca, Anna-Carin Olin, Håkan Tinnerberg

**Affiliations:** ^1^Occupational and Environmental Medicine, School of Public Health and Community Medicine, Institute of Medicine, University of Gothenburg, Gothenburg, Sweden; ^2^Occupational and Environmental Medicine, Sahlgrenska University Hospital, Gothenburg, Sweden; ^3^RISE, Research Institutes of Sweden, Gothenburg, Sweden; ^4^Occupational and Environmental Medicine Center in Linköping, and Department of Health, Medicine and Caring Sciences, Linköping University, Linköping, Sweden

**Keywords:** additive manufacturing, exposure, nanoparticles, exhaled air, lung surfactant, phosphatidylcholine

## Abstract

Additive manufacturing (AM), or 3D printing, is a growing industry involving a wide range of different techniques and materials. The potential toxicological effects of emissions produced in the process, involving both ultrafine particles and volatile organic compounds (VOCs), are unclear, and there are concerns regarding possible health implications among AM operators.

The objective of this study was to screen the presence of respiratory health effects among people working with liquid, powdered, or filament plastic materials in AM.

**Methods:**

In total, 18 subjects working with different additive manufacturing techniques and production of filament with polymer feedstock and 20 controls participated in the study. Study subjects filled out a questionnaire and underwent blood and urine sampling, spirometry, impulse oscillometry (IOS), exhaled NO test (FeNO), and collection of particles in exhaled air (PEx), and the exposure was assessed. Analysis of exhaled particles included lung surfactant components such as surfactant protein A (SP-A) and phosphatidylcholines. SP-A and albumin were determined using ELISA. Using reversed-phase liquid chromatography and targeted mass spectrometry, the relative abundance of 15 species of phosphatidylcholine (PC) was determined in exhaled particles. The results were evaluated by univariate and multivariate statistical analyses (principal component analysis).

**Results:**

Exposure and emission measurements in AM settings revealed a large variation in particle and VOC concentrations as well as the composition of VOCs, depending on the AM technique and feedstock. Levels of FeNO, IOS, and spirometry parameters were within clinical reference values for all AM operators. There was a difference in the relative abundance of saturated, notably dipalmitoylphosphatidylcholine (PC16:0_16:0), and unsaturated lung surfactant lipids in exhaled particles between controls and AM operators.

**Conclusion:**

There were no statistically significant differences between AM operators and controls for the different health examinations, which may be due to the low number of participants. However, the observed difference in the PC lipid profile in exhaled particles indicates a possible impact of the exposure and could be used as possible early biomarkers of adverse effects in the airways.

## 1. Introduction

3D printing, a type of additive manufacturing, is used in many different settings, including large-scale applications for commercial use, home-based businesses or hobby activities, university laboratories, and schools. The technique is ideal for producing complex structures, small-scale production, or prototypes and is currently a global growing market.

Methods involving additive manufacturing of polymer materials include either melting thermoplastic and depositing it on a plate or by sintering a powder bed, layer-by-layer, or using thermosetting liquid resin cured into layers. Some of the most used thermoplastic feedstocks are acrylonitrile butadiene styrene (ABS), polylactic acid (PLA), polyethylene terephthalate with glycol (PET-G), and polyamide (PA). The thermosetting liquid resins are usually epoxy-or acrylate-based. The techniques and pre-and post-related activities are described in a recent review by Stefaniak et al. (1).

The potential toxicological effects of emissions from 3D printing are unclear. Previous studies on emissions from polymer AM have shown that emissions are complex mixtures as can be expected from the combustion of polymer-based materials. Depending on technique and material, predominately ultrafine particles (<100 nm) are emitted in high concentrations, especially using material extrusion techniques (1–3). Isocyanic acid and volatile organic compounds (VOCs) are also emitted, including acetaldehyde, acetone, acrolein, formaldehyde, benzaldehyde, butadiene, iso-butanol, cyclohexanone, styrene, and ethylbenzene (3, 4).

It is difficult to predict the long-term health effects due to the complex nature of emissions. Acute exposure to VOC can lead to irritations in the eyes, nose, and throat as well as headache and nausea, and there is a risk factor of developing pulmonary diseases, including asthma and lung cancer after chronic/repeated exposure. Inhaled particles are either removed by mucociliary clearance or deposited in the lining fluid of the lungs where they may become opsonized and eliminated by macrophages. If they are in the nano-size, they can also be absorbed into the bloodstream (5–7). Polymer-based particles are generally considered to have low toxicity but may cause inflammation depending on the structure (e.g., fiber-like) and dose. Furthermore, additives in the particles may enhance the inflammatory response.

*In vitro* studies and animal studies have shown that exposure to ABS emissions from 3D printers based on extrusion techniques can cause systemic and local toxic effects involving oxidative stress and inflammatory responses (8–11). There are only a few studies on the health effects on humans and the use of 3D printers. A survey including workers in companies using 3D printers found that working more than 40 h per week with 3D was significantly associated with respiratory symptoms (12). Some individual cases have been described showing the risk of developing asthma from working with ABS (13) and contact dermatitis from working with epoxy resins (14). In a chamber study, Gümperlein et al. observed a small increase in exhaled nitric oxide after exposure to ABS compared to PLA (15). Würzner et al. studied the cytokine content in nasal secretions and lung diffusing capacity in individuals with seasonal allergic rhinitis exposed to 3D printer emissions from PLA and ABS and found small reductions in lung diffusion capacity for inhaled nitric oxide but overall no strong evidence for effects after the short-term exposure based on nasal allergen responses (16).

There is thus scarce knowledge on health issues related to the release and long-term exposure of nanoparticles from AM as well as material extrusion. Besides detailed exposure assessment, health surveillance can be a useful tool to assess and follow up on possible health implications on a group level as well as on an individual level. To this end, non-invasive methods such as the analysis of particles in exhaled air are attractive seeing that they are easy to perform and enable screening of large groups. The composition of endogenous particles in exhaled air reflects the composition of the respiratory tract lining fluid in the small airways and may therefore provide valuable information on the early effects of nanoparticle exposure (17, 18).

The primary aim of this study was to investigate the respiratory health among operators, working with polymeric AM and extrusion-based filament production, by traditional lung function measurements as well as the collection and analysis of exhaled air. In addition, clinical blood and urine markers including systemic markers of inflammation, cardiovascular disease, and renal and hepatic function were included.

## 2. Materials and methods

### 2.1. Exposure measurement strategy

Parts of the exposure measurements (PBF, VP, MJ, and parts of FDM) from this study have previously been published in detail, see ref. (3). Particulate and gaseous emissions were measured from four different additive manufacturing techniques and a number of different printers that were manufactured by three different companies. A pre-visit including walk-along sessions with working personnel was performed before the week of measurements. To give a good representation of the exposure, all measurements were performed within the same week as health assessments were performed. Health assessments were performed at the end of the week of exposure measurements, see Section 2.4.

Personal measurements for inhalable dust and complementary measurements of ultrafine particles as well as long-term measurements using particle sensors were performed for all techniques. An exception was made for printing with the VP technique where no inhalable fraction measurements were performed. For the powder-based technique (PBF), personal exposure to respirable dust was also measured. Nanoparticles were measured using a handheld particle counter, and measurements were performed close to the AM operators to identify tasks associated with high emissions. The particle sensors used were mounted close to the printers or at a nearby workspace, for 4–10 days, to give a more gathered view of the variation in emissions.

For VOC, personal exposure measurements were performed at all facilities and only during separate activities related to AM, e.g., cleaning, changing of feedstock, maintenance, or post-processes such as sanding of printed detail or removing of excess powder. Personal measurements were between 2 and 61 min, depending on the type of activity. Furthermore, in some facilities, little time was spent close to the printer, i.e., the printer and office spaces were in separate rooms and for these locations, stationary measurements were taken for VOCs close to the printers to give information regarding concentrations therein. Stationary measurements were between 30 and 69 min.

Moreover, isocyanates were measured when printing with polyurethane-containing material, see ref. (3) for details.

### 2.2. Manufacturing techniques and feedstock materials

The printing techniques included in this study were selective laser sintering (SLS)/powder bed fusion (PBF; Eosint P350 and Eosint 700), PolyJet/material jetting (MJ; Stratasys Connex 500 and Stratasys Eden 500), vat photopolymerization (VP)/stereolithography (SLA; Formlabs Form 2) and material extrusion (FDM or ME). Emissions were assessed from different methods of ME, both desktop fused deposit modeling (FDM; Original Prusa, Creator Pro, Zyyx Pro, Ultimaker 3, and Ultimaker 5) and industrial size FDM (Stratasys F370) as well as large-scale extrusion for the production of filament (ME). PBF, FDM, and ME are techniques based on the melting of polymer material (powder, granules, or filament), while MJ and VP are based on UV curing of an acrylate-based resin. All printers were closed industrial printers with process ventilation except for the desktop FDM printers and the extrusion process. However, change in feedstock, washing, sanding, and de-powdering were performed manually and therefore a potential source of exposure to both VOCs and particles.

Feedstock material for the different techniques was as follows; PBF: polyamide (PA12), MJ: acrylate-based liquid (VeroGray), vat photopolymerization: acrylate-based liquid (Blackv4), and FDM/ME: acrylonitrile butadiene styrene (ABS), thermo polyurethane (TPU), polylactic acid (PLA), and polyamide (PA) with and without carbon fiber reinforcement.

### 2.3. Measurements of dust, particles, and gaseous compounds

The methods used are described in detail in ref. 3. Briefly, high-time resolution particle emissions were measured using two different condensation particle counters (CPC): P-Trak 8,525 and Condensation Particle Counter 3,007 (TSI Incorporated, Minnesota 55,126 USA). The P-Trak 8,525 measures the number of particles in the size range of 20 nm-1 μm, whereas the CPC 3007 measures the number of particles in the size range of 10 nm-1 μm; none of these instruments can discriminate between different particle sizes in the range. These instruments were used at all facilities. For long-term measurements, a particle sensor such as Alphasense OPC-N3, measuring the number of particles in the size range of 0.35–40 μm, was used. Inhalable dust was measured with an IOM sampler (flow rate 2 l/min) and respirable dust with SKC respirable cyclone (2.5 l/min). Samplers were placed in the breathing zone of the operator. In general, these measurements were performed during a full workday (minimum 6 h).

Adsorbent tube VOC and TVOC measurements were performed according to the ISO 16000-6:2011 method. For details, see ref. (3).

### 2.4. Health examinations

Health examinations were performed on 18 AM polymer operators from the three different companies and 20 controls. Controls were recruited from the same companies as well as from an additional company that was included in a larger study also involving AM metal operators (not included in this study). Inclusion criteria for the controls included the absence of occupational exposure to processes that may generate airborne irritants. All examinations, except urine sampling (see details in Section 2.4.6), were performed at the same time point at the end of the work week (either Thursday or Friday) and included blood sampling, filling out a questionnaire, lung function measurement, impulse oscillometry (IOS), fraction of exhaled NO (FeNO), and sampling of particles in exhaled air. The participants did not have any restrictions regarding the medication before the examinations. The study protocol was approved by the Swedish Ethical Review Authority, no 2019–03536. All participants gave their oral and written informed consent.

#### 2.4.1. Questionnaire

All study subjects were asked to complete questionnaires regarding health conditions, work routines, smoking habits, and previous occupational exposure history.

#### 2.4.2. Lung function

Spirometry was performed with a JAEGER MasterScreen. The measurements were performed according to ATS/ERS recommendations (19). The forced expired volume in the first second (FEV1) and the forced expiratory vital capacity (FVC) were measured, and the ratio FEV1/FVC was calculated. Predicted normal values were based on local reference values from Brisman et al. (20).

#### 2.4.3. Impulse oscillometry

Impulse oscillometry (IOS) was measured with an IOS JAEGER MasterScreen or Vyntus IOS device according to ATS/ERS recommendations (21). Calibration was performed daily. The measurements were performed in triplicate.

#### 2.4.4. Fraction of exhaled nitric oxide

Fraction of exhaled NO (FeNO) was measured with a chemiluminescence nitric oxide analyzer (NIOX VERO® instrument AER-12-1850, Aerocrine AB, Stockholm, Sweden) at an expiratory flow of 50 ml/s. The measurements were in accordance with the recommendations made by the American Thoracic Society (ATS) and the European Respiratory Society (ERS) (22). One AM operator did not perform the FENO test.

#### 2.4.5. Collection and analysis of particles in exhaled air (PEx)

The collection of particles in exhaled air (PEx) was performed using the PExA 2.0 instrument (PExA, Gothenburg, Sweden). All study participants performed an airway reopening breathing maneuver inhaling particle-free air and exhaling into the instrument by means of a two-way valve. An optical particle counter recorded particle number concentration and particle number size distribution (0.41–4.55 μm). Particles were collected on a polytetrafluoroethylene (PTFE)-membrane which was split into two equal parts after sampling. A minimum of 80 ng of exhaled particles was collected from each individual. The split membrane samples were stored in separate tubes at −80°C until analysis.

The enzyme-linked immunosorbent assay (ELISA) technique was used to quantify the specific proteins of interest in PEx. The manufacturer’s instructions for SP-A-ELISA (BioVendor, Brno, Czech Republic) and albumin ELISA (E-80AL Immunology) were applied with minor changes. PEx samples were extracted from PTFE-membranes using 140 μl of extraction buffer with a composition of 10 mM PBS containing 1% BSA w/v and 0,05% TWEEN-20 v/v (Thermo Scientific, Rockford, IL, USA) for 60 min using a thermomixer set at 37°C and 400 rpm (Comfort, Eppendorf AG, Hamburg, Germany). The extracted sample volume was aliquoted into three different vials and stored at −20°C until analysis: 40 μl for SP-A and albumin and the rest as a backup. Prior to analysis, 80 μl of assay diluent buffer was added to the samples. A dilution buffer (extraction buffer:assay diluent buffer, 1:2 ratio) was prepared for the reconstitution of standards and controls to match the sample buffer composition. Samples were incubated for 2 h at 37°C with shaking at 300 rpm. Albumin was incubated for 1 h at room temperature with shaking at 300 rpm. Reaction time was 9 min. The precision of the assays was monitored by three sample duplicates in each run. The Coefficient of variation (CV) of the duplicates for SP-A and albumin was 1.1–6.8% with no significant difference between the two assays.

For the analysis of phosphatidylcholine (PC) lipids, PEx samples, quality control samples (diluted bronchoalveolar lavage), and blank PEx membranes were extracted in two steps: (1) 200 ul of methanol:chloroform (50:50) was added to the vial with the membrane, followed by shaking for 5 min, and transferal of the solvent to a glass evaporation vial and (2) adding 200 ul of methanol to the vial with the membrane, followed by vortexing, and transferal to the glass evaporation vial. Samples were dried under N_2_. The samples were then redissolved in 60 μl of ACN:H2O (95:5) and transferred to new glass vials. The extracted samples were stored at −20°C until analysis, which was performed within 1 month. Organic solvents were of LC–MS grade (Merck KGaA, Darmstadt, Germany). Ammonium acetate was purchased from Sigma Aldrich (St. Louis, MO, USA).

Samples were run on a Waters Acquity UPLC I-Class PLUS system coupled with a XEVO TQ-XS mass spectrometer with an electrospray ionization source operating in multiple reaction monitoring modes (MRM). Chromatographic separation was performed on a Waters Acquity UPLC BEH C8 column (2.1 × 100 mm, 1.7 μm). The gradient mobile phase was composed of A: water/acetonitrile (90:10, v/v) with 10 mM ammonium acetate and B: acetonitrile/isopropanol/water (70, 20:10, v/v/v) with 10 mM ammonium acetate. The flow rate was 0.6 ml/min, and the column temperature was 50°C. Initial conditions were 9% A and 91% B, followed by a linear gradient from 91 to 100% B within x min, after which 100% B was held for 1.5 min, followed by re-equilibration for 1.5 min. MS/MS scans were performed by 35 negative MRM scans, summarized in Supplementary Table 1. MS conditions were as follows: capillary voltage 1 kV, cone voltage 25 V, desolvation temperature 600 C, desolvation gas flow 1,000 l/h, cone gas flow 150 l/h, collision energy 40 eV, and cone voltage 15V. MassLynx V4.2 and TargetLynx XS were used for data acquisition and processing. Samples were run in a randomized order. High-and low-quality control samples (n = 3) were run before, between, and after samples. Lipid signals with CV >20% for the corresponding control samples were excluded from the final analysis. Molecular PC lipid signals were normalized to the total PC lipid signal.

#### 2.4.6. Urine sampling and analysis

Participants collected a spot sample of morning urine at the start and the end of the workweek. Alpha-1-microglobulin (U-α1 M), which is a clinical marker for renal function, was analyzed at Clinical Chemistry Laboratory, Linköping University Hospital, Sweden. Linkoping. Levels were creatinine adjusted. One AM worker did not leave urine samples.

#### 2.4.7. Blood sampling and analysis

Blood was sampled during the day at the end of the workweek in lithium heparin tubes, centrifuged at 1200 *G* for 12 min, and plasma was transferred to new tubes and frozen at −20°C until analysis.

Hepatic function markers aspartate aminotransferase (ASAT), alanine aminotransferase (ALAT), and alkaline phosphatase (ALP) as well as markers for cardiovascular disease, apolipoprotein B (ApoB), and apolipoprotein A1 (ApoA1) were analyzed at Clinical Chemistry Laboratory, Linköping University Hospital, Sweden. Analyses of the inflammatory marker acute phase proteins serum A (SAA) and the antioxidant activity protein paraoxonase-1 (PON1) were performed by ELISA, and an in-house enzyme activity assay previously described in ref. 23.

### 2.5. Statistical analysis of clinical parameters

Based on the results here and previous studies (2), FDM and ME are techniques associated with relatively high nanoparticle emissions compared to the other techniques and were therefore of interest to look at separately. In addition, both are extrusion techniques using thermoplastic feedstock. Hence, in addition to comparing all AM operators with the control group, a subgroup composed of operators working exclusively with material extrusion techniques ME and/or FDM was also compared to the control group.

The Wilcoxon rank-sum test (Mann–Whitney *U*-test) was performed to explore the differences between the groups using SAS 9.4. Tests were two-tailed, and the results were considered statistically significant when the *p*-value was <0.05. In addition, to detect potential outliers as well as to visualize patterns between groups, a principal component analysis (PCA) was performed for PC lipids in PEx using SIMCA 17. PCA is a dimension-reduction statistical method suitable for data with a high level of correlation between variables and for identifying underlying structures, grouping, and patterns.

## 3. Results

### 3.1. Exposure and emission measurements

#### 3.1.1. Dust and particle measurements

Inhalable dust levels were below or just at a detection level of 90 μg (corresponding to <180 μg/m^3^) for FDM, ME, and MJ. Samples from the PBF technique were, however, all above detection levels, but still below the Swedish occupational exposure limit (5 mg/m^3^), ranging from 0.1 to 1.9 mg/m^3^ (3).

The highest particle numbers were seen in facilities with insufficient ventilation and an open extrusion process (FDM and ME) with background concentrations of 50–100,000 particles/cm^3^ and with emissions from the process with concentrations above 500,000 particles/cm^3^ (maximum output of measuring instrument).

Fused deposit modeling printers within other facilities had either local exhaust ventilation or were encapsulated with filters, giving low particle emissions (<10,000 particles/cm^3^). For comparison, and to gather a view of concentrations without these measures to mitigate the exposure, samples were taken within the print chambers showing notably higher concentrations depending on the time from print start (7000–480,000 particles/cm^3^) (3).

Liquid-based printing techniques, i.e., MJ and VP produced the lowest particle emissions during print and post-processes (<5,000 particles/cm^3^; details see ref. (3)).

#### 3.1.2. VOC measurements

The results from the VOC measurements also showed a large variation in the emission of total volatile organic compounds (TVOCs) as well as in identified individual VOC species depending on the print technique, material used, and design of the facilities. Generally, low TVOC concentrations, compared to the background, were seen for FDM and VP prints (~100 μg/m^3^) and higher for MJ (~3,000 μg/m^3^). The highest concentrations of TVOC were, however, measured during the maintenance work of the PBF printer, see ref. (3).

**Figure 1:**
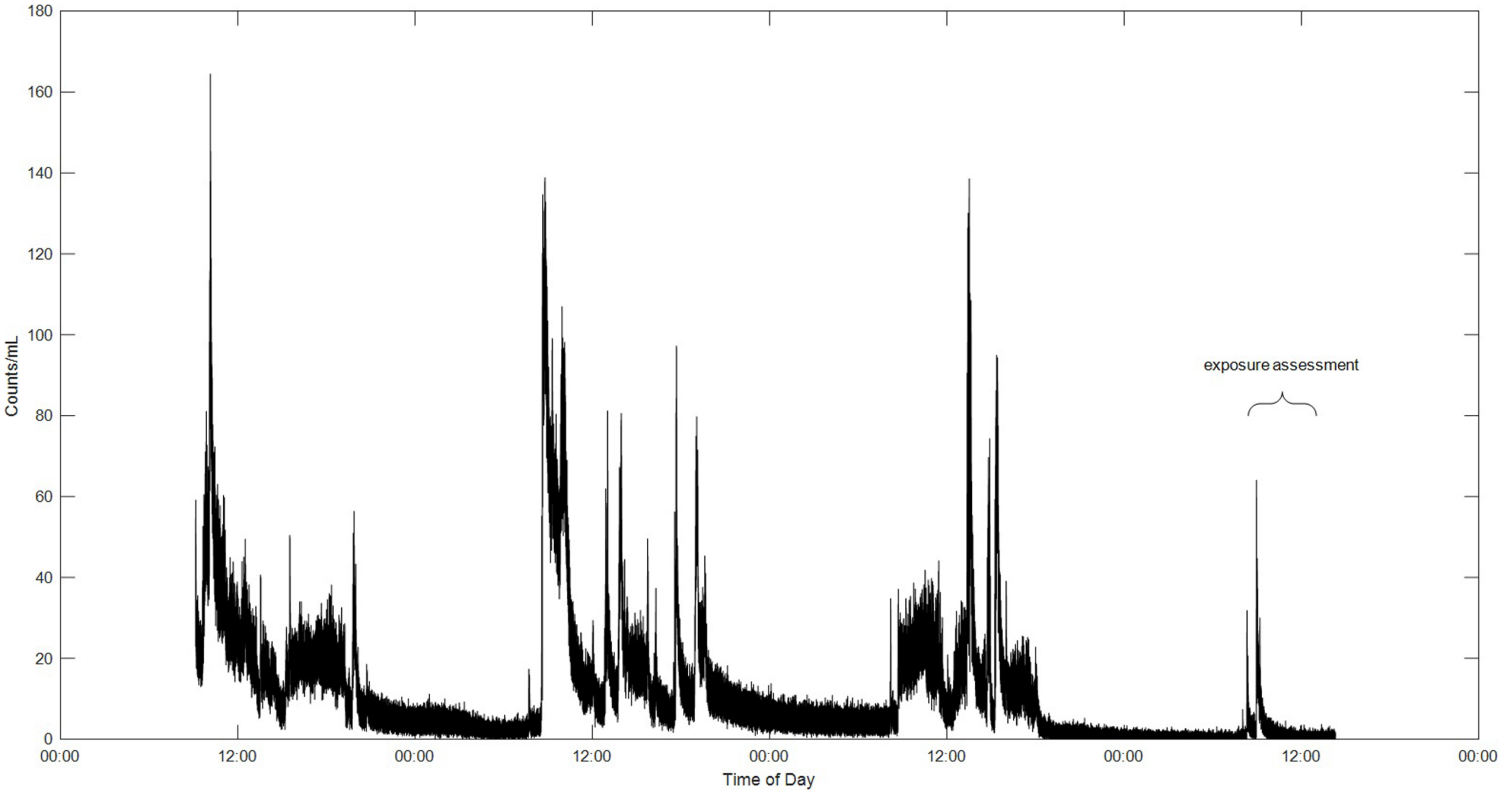
Data from a particle sensor placed at a workspace, in the same facility as the extrusion process, for four workdays showing the fluctuations of emissions during the week.

Sensors, both particle and VOC, placed in the facilities indicated large variations in emissions (see Figure 1 for particles), also within the facilities, throughout the workweek, indicating that exposure for personnel working in the field of additive manufacturing is difficult to estimate.

#### 3.1.3. Identification of individual VOCs

Individual VOC species were identified and quantified from all four polymer AM techniques; PBF, VP, FDM, and MJ; see ref. (3) and Supplementary Table 2.

In general, aliphatic hydrocarbons and aldehydes dominated prints with the FDM technique along with specific species depending on the feedstock, e.g., lactide (from PLA), styrene (from ABS), and caprolactam (from PA). Techniques using liquid resin and UV curing, MJ and VP, show overall higher concentrations than other printing techniques mainly due to washing with isopropanol and acrylates dominating in the feedstock. All compounds detected either lacked or were well below their occupational exposure limits.

#### 3.1.4. Exposure assessment

In this study, personal exposure measurements and stationary measurements along with sensor measurements were performed. A gathered exposure assessment was, however, not feasible due to the large variation between individuals in factors such as printer technique, time spent by printer, print material, and occurrence of preventive measures. For some facilities, separate print rooms and office spaces decreased the risk of exposure substantially, while others had desktop printers at the workstation or lacked sufficient ventilation causing contamination of the office space air. No personal protective equipment such as respiratory masks was used in these facilities.

### 3.2. Health examinations

The results of the health examinations are presented for all polymeric AM operators (including FDM and ME) as well as for the subgroup of AM operators operating only FDM printers and/or ME and for the controls. Characteristics of all participants are presented in Table 1.

**Table 1:** Characteristics of additive manufacturing (AM) subjects and controls in the study.

	AM operators		Controls *n* = 20
	All (*n* = 18)	FDM/ME (*n* = 10)	
Sex (f/m)	1/17	1/9	7/13
Age (years)	33 (31, 43)	33 (31, 42)	40 (36, 46)
BMI (kg/m^2^)	25.4 (23.0, 31.4)	24.6 (22.5, 28.1)	24.4 (22.1, 25.9)
Smoking
Current smoker (“do you smoke every day?”)	0	0	0
Occasional smoker (“do you smoke at parties?”)	5	2	1
Former smoker	3	2	3
Never smoked	10	6	16
Physician-diagnosed asthma (yes/no)	3	1	1
Prevalence of respiratory symptoms for the past 12 months
Nose problems*	6	3	8
Dry cough	2	0	1
Trouble with breathing	0	0	0

Medians and interquartile range are listed.

* Nasal congestion, runny nose, or sneezing without having a cold.

#### 3.2.1. Questionnaire

Additive manufacturing operators did not report any higher prevalence of either upper or lower respiratory symptoms such as nose problems, coughing, or wheezing compared to the control group. None of the AM operators reported any trouble with breathing for the past 12 months (see Table 1). Three individuals reported having physician-diagnosed asthma but also reported having no asthma symptoms for the past 12 months, and the debut of asthma was before working with AM. Two of them were on asthma medication (corticosteroids). Among the controls, one individual reported having physician-diagnosed asthma.

#### 3.2.2. Lung function, FeNO, IOS, and PEx

All AM operators had normal lung function based on spirometry variables. There were also no statistically significant differences between AM operators and controls. There were no statistically significant differences between the groups regarding FeNO levels or IOS variables (see Table 2).

**Table 2:** Results of the lung function tests and breath analyses.

	AM operators		Controls (*n* = 20)
	All (*n* = 18)	Only FDM/ME (*n* = 10)	
Spirometry
FEV1%pred	103 (93, 112)	101 (92, 110)	99 (84, 105)
FVC %pred	103 (96, 111)	102 (100, 111)	102 (96, 108)
FEV1/FVC %pred	99 (96, 103)	99 (94, 99)	98 (93, 103)
FeNO (ppb)	18 (15, 25)	16 (12, 25)	18 (13, 22)
IOS
IOS R5 (Hz) %pred	105 (88, 119)	102 (93, 127)	106 (83, 111)
IOS R20 (Hz) %pred	112 (94, 123)	117 (101, 123)	108 (89, 129)
IOS R5-R20 (Hz)	0.02 (0.01, 0.06)	0.02 (0.01, 0.04)	0.02 (0.01, 0.06)
IOS AX (Hz·kPa·L-1)	0.13 (0.09, 0.28)	0.12 (0.10, 0.13)	0.16 (0.13, 0.56)
PEx
PEx number/breath	22,000 (13,500, 72,100)	17,500 (12,700, 38,500)	37,300 (24,300, 68,300)
PEx SP-A (%PEx)	2.9 (2.7, 3.6)	2.9 (2.7, 3.6)	3.3 (2.7, 3.9)
PEx Albumin (%PEx)	4.3 (3.6, 5.9)	4.5 (3.6, 5.9)	4.0 (3.5, 4.5)
PEx Albumin/SP-A	1.5 (1.1, 2.0)	1.6 (1.1, 2.0)	1.2 (1.0, 1.5)

Medians and interquartile ranges are listed. There were no significant differences between the groups.

There were no statistically significant differences between the groups regarding particle numbers per number of breaths (PEx number/breath, see Table 2). The levels of SP-A and albumin in exhaled particles were not statistically significantly different between the groups.

**Figure 2:**
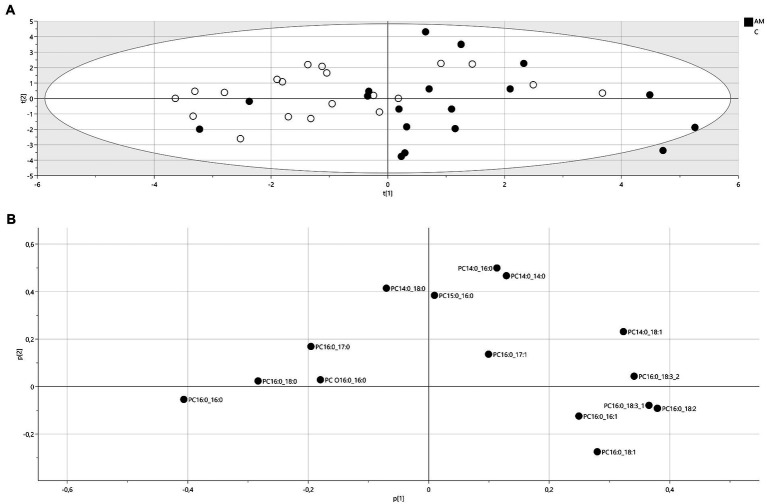
Principal component analysis **(A)** score plot of PC lipids (R2X(*cum*) = 0.575, Q2 (*cum*) = 0.282), filled circles: AM operators, unfilled circles: controls. t(1): first principal component, t(2):second principal component and **(B)** corresponding loadings plot. A variable with a positive loading is positively correlated to the principal component, and negative loadings indicate a negative correlation. PC: phosphatidylcholine, number of carbons:number of double bonds.

In total, 15 PC species were determined in all PEx samples. The PC composition was examined by PCA to visualize groupings and differences between the AM group and the control group (see Figure 2 for the PCA score and corresponding loading plots). In the score plot, each spot represents one individual. The distance between spots in the score plot indicates similarities between individuals that are explained in the loading plot. Here, there was a pattern suggesting a difference in the abundance of unsaturated and saturated PC species between the groups. The Wilcoxon statistical test on the individual PC lipids confirmed this observation, including the saturated PC lipid dipalmitoylphosphatidylcholine (PC16:0_16:0) that was significantly higher among controls compared to both the groups with all AM operators as well as the group with AM operators using FDM/ME and unsaturated PC lipid palmitoyllinoleoylphosphatidylcholine (PC16:0_18:2) that was higher among both groups of AM operators compared to the control group (see Table 3).

**Table 3:** Medians and interquartile range (25–75%) of PCs in exhaled particles.

	AM operators		Controls (n = 20)
	All (n = 18)	Only FDM/ME (n = 10)	
PC 14:0_14:0	0.003 (0.002, 0.006)	0.005 (0.002, 0.006)	0.004 (0.003, 0.005)
PC 14:0_16:0	0.085 (0.069, 0.095)	0.089 (0.069, 0.117)	0.089 (0.078, 0.102)
PC 15:0_16:0	0.016 (0.014, 0.019)	0.018 (0.015, 0.020)	0.017 (0.016, 0.021)
PC O-16:0_16:0	0.001 (0.001, 0.002)*	0.002 (0.001, 0.002)	0.002 (0.002, 0.002)
PC 16:0_16:1	0.100 (0.084, 0.122)	0.106 (0.094, 0.131)	0.097 (0.079, 0.105)
PC 14:0_18:1	0.009 (0.007, 0.011)*	0.010 (0.008, 0.012)*	0.007 (0.006, 0.009)
PC 14:0_18:0	0.002 (0.001, 0.003)	0.001 (0.001, 0.003)	0.002 (0.002, 0.002)
PC 16:0_16:0	0.533 (0.520, 0.583)*	0.525 (0.516, 0.531)*	0.578 (0.547, 0.618)
PC 16:0_17:1	0.004 (0.004, 0.005)	0.005 (0.004, 0.006)	0.004 (0.004, 0.005)
PC 16:0_17:0	0.012 (0.009, 0.013)	0.012 (0.010, 0.013)	0.012 (0.011, 0.014)
PC 16:0_18:3	0.003 (0.002, 0.004)*	0.003 (0.002, 0.004)	0.002 (0.002, 0.003)
PC 16:0_18:3	0.002 (0.002, 0.003)	0.002 (0.002, 0.003)	0.002 (0.001, 0.002)
PC 16:0_18:1	0.149 (0.127, 0.161)*	0.149 (0.135, 0.161)*	0.130 (0.116, 0.139)
PC16:0_18:0	0.008 (0.006, 0.009)	0.007 (0.006, 0.008)*	0.010 (0.007, 0.011)
PC 16:0_18:2	0.049 (0.046, 0.073)*	0.050 (0.048, 0.076)*	0.040 (0.032, 0.050)

Molecular PC lipids are expressed as relative ratios of the total PC lipid signal. *Significantly different from the control group, *p* < 0.05. PC, phosphatidylcholine, number of carbons:number of double bonds.

#### 3.2.3. Blood and urine parameters

The levels of clinical markers in blood and urine are presented in Table 4. All the levels were within clinical chemistry reference values. On the group level, the levels of ApoA1 were statistically significantly decreased among AM operators compared to the control group. This decrease was also observed among AM operators working with FDM/ME.

**Table 4:** Results from the clinical blood and urine analyses.

	AM operators		Controls (*n* = 20)
	All (*n*=18)	Only FDM/ME (*n* = 10)	
ASAT (μkat/L)	0.49 (0.37, 0.69)	0.47 (0.37, 0.50)	0.41 (0.36, 0.50)
ALAT (μkat/L)	0.40 (0.24, 0.49)	0.35 (0.23, 0.45)	0.26 (0.22, 0.41)
ALP (μkat/L)	1.02 (0.77, 1.14)	1.02 (0.82, 1.09)	0.88 (0.78, 1.13)
ApoA1 (g/L)	1.36 (1.29, 1.44)	1.36 (1.29, 1.44)*	1.45 (1.35, 1.71)
ApoB (g/L)	0.96 (0.86, 1.09)	0.87 (0.81, 1.00)	0.84 (0.73, 1.10)
ApoB/ApoA1	0.71 (0.62, 0.79)	0.66 (0.52, 0.78)	0.61 (0.44, 0.74)
SAA1/PON1 (ng/U)	15.2 (9.11, 22.8)	18.6 (9.11, 22.6)	9.26 (7.39, 15.9)
U-α1M (mg/L)	0.47 (0.32, 0.73)	0.55 (0.39, 0.73)	0.4 (0.23,0.54)

Medians and interquartile ranges are listed. *Significantly different from the control group, *p* < 0.05.

## 4. Discussion

An increasing number of exposure studies in AM settings have presented results on significant emissions of ultrafine particles and VOCs, both of which may affect respiratory health upon inhalation. There are, however, to date no studies addressing both operators’ exposure and possible effects on health in real settings. In the present study, exposure assessment and screening of AM operators recruited from different companies were performed. In total, seven different print facilities operating different AM printing and/or extrusion techniques were included. Production of filaments from polymer pellets by extrusion was here considered to give similar exposure as 3D printing of the same material. All participants underwent blood and urine sampling, lung function tests, and a sampling of particles in exhaled air.

A major challenge regarding exposure and risk assessment in AM environments is the wide range of different techniques and materials used. An additional challenge in this context is that the heating and combustion of these materials result in the emission of gas and vapor mixtures with a complex composition that depends on both the chemical components of the raw material and the temperature. In addition to the large variation in printing techniques and materials used, the workload and the design of the printing facilities (including ventilation solutions) also varied greatly, and thus, a gathered exposure for AM workers, both on the individual level and group level was difficult to assess. Most of the operators in this study worked with thermoplastic materials such as PA (PBF and FDM), ABS, TPU, and PLA (FDM) but some individuals also operated techniques with thermoset materials (3).

Furthermore, the concentration and content of particles and gases vary within printing operating time and printer make where an increase in particle number often occurs at the beginning of the print but may also accumulate in the facilities if the ventilation is insufficient. In this study, we were able to measure particles and gases while printing with all techniques and materials tested even when encapsulation and ventilation were used as preventive measures. The results presented herein showed that groups of organic compounds, e.g., acrylates, aliphatic hydrocarbons, aldehydes, and alcohols were present in emissions from feedstock and maintenance work. Techniques giving rise to the highest levels of VOC and compounds identified as dominating, e.g., methyl methacrylate (VP) and isobornyl acrylate (MJ), show a good correlation with a previous study of VOCs from various print techniques performed by Väisänen et al. (2).

Inhalable dust levels were below, or just at, the detection limit for all measurements performed during FDM print and extrusion, but on the other hand, considerable amounts of nanoparticles were detected using condensation particle counters. More than 10 times the concentration (180,000–500,000 particles/cm^3^) was found when measurements were performed close to the source. Broekhuizen et al. suggested a nano reference value of 40,000 particles/cm^3^ for nanoparticles with a density below 6 g/cm^3^ (polymer particles have a density of 1–2 g/cm^3^), which was exceeded in this study for all FDM and extrusion prints with no encapsulation or filter (23). Other techniques in this study, i.e., VP, PBF, and MJ, had concentrations below 20,000 particles/cm3 (PBF) or even below 5,000 particles/cm3 (VP and MJ), which is in agreement with other studies (24).

Seeing that FDM and ME are AM techniques that generate high nanoparticle concentrations and that most AM operators in this study used FDM or material extrusion, it was of interest to perform comparisons of health measurements in both all AM operators and in this subgroup (working exclusively with FDM and/or FDM/ME), despite varying exposures in the groups due to preventive measures, workload, and design of the facilities. Spirometry and IOS parameters and FeNO were within normal ranges for all AM operators, including the subgroup FDM/ME, and they did not differ from the control group, nor were there any differences between any of the groups regarding levels of FeNO. FeNO has been suggested as an early indicator of eosinophilic airway inflammation and asthma and a useful tool for medical surveillance in workplaces in non-smokers, in particular for exposure to high-molecular agents.

A major concern of exposure to airborne nanoparticles is their ability to reach and disturb surfactant structure and biophysical function and induce an inflammatory response in the peripheral airways and the alveolar space. Exposure to polystyrene nanoparticles was shown to inhibit the surface activity of surfactant *in vitro* (25) and has, depending on surface chemistry, been observed to cause interleukin-6 and interleukin-8 release in alveolar type I-like cells (26). Here, the analysis of exhaled particles revealed some differences between groups that may be related to the early alterations of the surfactant barrier. There was a decrease in the relative ratio of surfactant lipids dipalmitoylphosphatidylcholine (PC16:0_16:0) and palmitoylstearoylphosphatidylcholine (PC16:0_18:0; only FDM/ME group) and an increase in the relative ratio of palmitoyllinoleoylphosphatidylcholine (PC16:0_18:2) and palmitoyloleoylphosphatidylcholine (PC16:0_18:1) compared to the control group. Furthermore, PCA revealed a difference in the pattern of saturated and unsaturated lipids between the groups. The difference observed in PC composition may reflect a reduction due to nanoparticle adsorption but may also be related to inflammatory processes. An increase of PC lipids containing linoleic acid (18:2) in bronchoalveolar lavage has previously been linked to the infiltration of plasma lipoproteins due to lung inflammation (27). Other components of interest in exhaled particles are SP-A and albumin. SP-A is involved in the lung host defense mechanisms by enhancing the elimination of foreign particles and pathogens and has been suggested as a biomarker of lung disease and injury. Albumin in PEx has been suggested as a marker of pulmonary epithelial cell injury and membrane permeability (28). Farcas et al. measured SP-A and SP-D in BALF from rats exposed to ABS emissions from 3D printing and found slightly decreased levels, although not significantly, compared to non-exposed (10). Levels of SP-A in PEx have been studied in individuals with asthma with conflicting results (29–31). In this study, we did not observe any differences between groups regarding SP-A and albumin. The number of exhaled particles has also been proposed as a marker of airway disease. A decreased concentration of exhaled particles has previously been observed in individuals with chronic obstructive pulmonary disease and asthma (29, 31, 32). One suggested explanation is that chronically inflamed airways and airway obstruction lead to air trapping. Here, we did not see any differences between the studied groups.

Clinical blood and urine markers were all within reference ranges. However, ApoAI was significantly decreased among the AM workers compared to the control group which could be a possible sign of an effect related to exposure but is more likely because there were more female subjects in the control group. The reference interval ApoA1 is higher for women than for men.

Overall, this study highlights the diverse and complex exposure in AM settings and the possibilities that health surveillance may enable an early risk assessment when exposure assessment is difficult to perform and knowledge of the hazards, here of nanoparticle emissions, is unknown. Routine clinical measurements such as spirometry are not sensitive enough to detect early changes and therefore new methods such as IOS and PExA have emerged as new tools to explore and evaluate. Here, we did on-site exposure measurements in several AM settings and included routine measurements including spirometry and determination of biomarkers in urine, blood as well as IOS, FeNO, and PExA to evaluate their usefulness in future follow-up studies regarding risk assessment in AM.

A main limitation is the relatively small number of exposed individuals that were included and that only one measurement per individual was performed. The included companies were either starting up AM activities (with the plan to scale up in near future) or were small-scale start-ups. From our experience, polymeric AM settings are often run by only a few individuals, making studies that include both exposure and health measurements challenging. Another limitation is the interpretation of the results from the analysis of exhaled particles. The possibilities of drawing any certain conclusions are limited based on the observed results. It is a relatively new method and there are thus far, few studies in an occupational context. Consequently, the full pattern of PC lipids in exhaled particles has not yet been fully explored and validated. Before planning the study, a sample size calculation was performed for DPPC based on previous (non-published) pilot studies, but no previous data was available for all lipids included here. Therefore, results must be interpreted with caution seeing that groups were small and that they were not matched in terms of, e.g., sex. Unknown sex-based differences in lipid and particle composition could be contributing to the results. These observations should be seen as an exploratory way of performing untargeted health surveillance as a complement to routine clinical tests. Nevertheless, the study supports the analysis of particles in exhaled air as a promising method in occupational settings, specifically concerning nanoparticle exposure, to detect early signs of surfactant alterations in the peripheral airways that, in turn, could be related to early signs of adverse effects. This tool along with other methods exploring small airway disease can be further explored for risk assessment in occupations where preventive measures are incomplete and risks unknown.

## 5. Conclusion

In this study, no statistically significant differences were observed for the different health examinations performed with well-established clinical methods, which could be due to the small number of participants. However, exploratory analysis of particles in exhaled air revealed a statistically significant difference between AM operators and controls in the lung surfactant composition that may be related to occupational exposure.

Analyzing particles in exhaled air is a promising way to surveil the health effects of nanoparticle exposure in AM environments and should be further explored and validated in longitudinal health surveillance studies.

## Data availability statement

The raw data supporting the conclusions of this article will be made available by the authors, without undue reservation.

## Ethics statement

The studies involving human participants were reviewed and approved by Swedish Ethical Review Authority. The patients/participants provided their written informed consent to participate in this study.

## Author contributions

A-CA, AB, HK, MA, A-CO, and HT contributed to conception and design of the study. AB and GR performed exposure measurements. A-CA, HK, and MA assisted in sample collection. A-CA, AB, HKo, and MA performed the chemical analyses. A-CA wrote the first draft of the manuscript. AB, HKo, and HT wrote sections of the manuscript. All authors contributed to the article and approved the submitted version.

## Funding

The study was funded by a grant from the Swedish Innovation Agency (Vinnova), HÄMAT2 (dnr 2018-03336), and the Swedish Research Council for Health, Working Life and Welfare (Forte) (dnr 2018-00290).

## Conflict of interest

The corresponding author, A-CA and A-CO are shareholders of PEXA AB and A-CO is a board member.

The remaining authors declare that the research was conducted in the absence of any commercial or financial relationships that could be construed as a potential conflict of interest.

## Publisher’s note

All claims expressed in this article are solely those of the authors and do not necessarily represent those of their affiliated organizations, or those of the publisher, the editors and the reviewers.Any product that may be evaluated in this article, or claim that may be made by its manufacturer, is not guaranteed or endorsed by the publisher.
