# Supplementary material for: An explorative study on respiratory health among operators working in polymer additive manufacturing
Source: Front Public Health. 2023 Apr 17;11:1148974. doi: 10.3389/fpubh.2023.1148974 (PMC10155750; doi:10.3389/fpubh.2023.1148974)
Supplement: Supplementary file 1 [file Table_1.docx]

Supplementary tables

**Table 1** List of all analyzed phosphatidylcholine species and corresponding retention times and MRM transitions. PC: phosphatidylcholine and fatty acid composition (number of carbons: number of double bonds).

| Name | Retention time (min) | Precursor ion  [M+59]^-^ | Product ion |
| --- | --- | --- | --- |
| PC14:0_14:0 | 1.47 | 736.5 | 227.3 |
| PC14:1_16:0 | 1.56 | 762.5 | 225.3 |
| PC14:0_16:1 | 1.55 | 762.5 | 253.2 |
| PC14:0_16:0 | 1.86 | 764.5 | 227.3 |
| PC16:1e_16:0 | 2.55 | 776.6 | 255.2 |
| PC15:0_16_0 | 2.07 | 778.6 | 241.2 |
| PC O-16:0_16:0 | 2.65 | 778.6 | 255.2 |
| PC16:1_16:1 | 1.62 | 788.5 | 253.3 |
| PC14:0-18:2 | 1.61 | 788.5 | 279.3 |
| PC16:0_16:1 | 1.94 | 790.6 | 253.3 |
| PC14:0_18:1 | 1.94 | 790.6 | 281.3 |
| PC14:0_18:0 | 2.35 | 792.6 | 227.3 |
| PC16:0_16:0 | 2.33 | 792.6 | 255.3 |
| PC15:0_18:1 | 2.16 | 804.6 | 241.2 |
| PC16:0_17:1_1 | 2.18 | 804.6 | 267.2 |
| PC16:0_17:1_2 | 2.40 | 804.6 | 267.2 |
| PC16:0_17:0 | 2.55 | 806.6 | 269.2 |
| PC16:0_18:3_1 | 1.70 | 814.6 | 277.3 |
| PC16:0_18:3_2 | 1.81 | 814.6 | 277.3 |
| PC16:1_18:2 | 1.68 | 814.6 | 279.3 |
| PC16:1_18:1 | 2.03 | 816.6 | 253.3 |
| PC16:0_18:2 | 2.02 | 816.6 | 279.3 |
| PC16:1_18:0 | 2.46 | 818.6 | 253.3 |
| PC16:0_18:1 | 2.44 | 818.6 | 281.3 |
| PC16:0_18:0 | 2.91 | 820.6 | 255.3 |
| PC16:0_20:5 | 1.68 | 838.6 | 301.2 |
| PC18:2_18:2 | 1.75 | 840.6 | 279.3 |
| PC16:0_20:4 | 1.98 | 840.6 | 303.2 |
| PC18:1_18:2 | 2.12 | 842.6 | 279.3 |
| PC18:0_18:2 | 2.56 | 844.6 | 279.3 |
| PC18:1_18:1 | 2.54 | 844.6 | 281.3 |
| PC18:0_18:1 | 3.05 | 846.6 | 281.3 |
| PC16:0_22:6 | 1.83 | 864.6 | 327.2 |
| PC18:1_20:4 | 2.06 | 866.6 | 303.2 |
| PC18:0_20:4 | 2.50 | 868.6 | 303.2 |

**Table 2** Dominating compounds emitted during printing are presented along with concentrations in background and during print for other techniques also see Runström et al (1)

| Technique/material | Compound | CAS nr | Concentration µg/m^3^ background | Concentration µg/m^3^ print |
| --- | --- | --- | --- | --- |
| FDM PLA  (personal sampling) | 3-methylpentane  n-hexane  Lactide | 96-14-0  110-54-3  4511-42-6 | 4  <2  2 | 11  9  11 |
| FDM ABS  (stationary sampling) | Isopropanol  Cyclopentane  Nonanal  Decanal | 67-63-0  287-92-3  124-19-6  112-31-2 | 200  15  <2  <2 | 430  11  3  3 |

1. Runstrom Eden G, Tinnerberg H, Rosell L, Moller R, Almstrand AC, Bredberg A. Exploring Methods for Surveillance of Occupational Exposure from Additive Manufacturing in Four Different Industrial Facilities. Ann Work Expo Health. 2022;66(2):163-77.
